# Erdosteine Therapy for Renal Failure: Current Perspectives

**DOI:** 10.5812/numonthly.2628

**Published:** 2012-06-20

**Authors:** Arunachalam Muthuraman

**Affiliations:** 1 Department of Pharmaceutical Sciences and Drug Research, Punjabi University, Patiala, India

**Keywords:** Erdosteine, Cyclosporine, Renal Insufficiency

Dear Editor,

I read the recently published paper of Ebru Uz et al. (1), in the Nephro-Urology Monthly which focused on the renoprotective effects of erdosteine when it is used for cyclosporine-A (CsA) induced chronic nephrotoxicity. Erdosteine belongs to the class of mucolytics which is used for the treatment of wet cough and chronic obstructive pulmonary disease (COPD). It has been reported that erdosteine possesses multiple mechanisms to protect against hepatic, renal, retinal and cardiotoxicity (2, 3). Erdosteine is a prodrug and metabolite I is the active metabolite of erdosteine owing to its free thiol group. It modifies the clinical outcome by improving the symptoms and reducing the progress of the disease. Pharmacokinetics of erdosteine results in rapid absorption after oral administration; distribution with protein binding is 64.5%; first pass metabolism to form an active metabolite i.e., N-thiodiglycolyl-homocysteine t_1/2_ 4.6 hour in rats; excretion through urine as metabolites, elimination half-life approximately 1.46 hr (erdosteine), and about 1.62 hr (metabolite). Common adverse reactions/side effects are epigastralgia, nausea, vomiting, diarrhea, spasmodic colitis, taste alteration, urticaria, eczema, erythema and headaches (4). Contraindications are, caution to be used during pregnancy and lactation. Erdosteine contains two sulfhydryl groups and it is reported that in the pharmacodynamics it breaks the disulphide bonds, which hold the glycoprotein fibers of mucus together. Furthermore, it makes the secretions and mucous more fluid which enhances elimination (5).

CsA is an immunosuppressant drug widely used in post-allogeneic organ transplants to reduce the activity of the immune system, and therefore it lessens the risk of organ rejection. CsA, was initially isolated from the fungus Tolypocladium inflatum and extracted from a soil sample, this was obtained by Sandoz scientists at Hardangervidda, Norway in 1969 (6). However, CsA is a very lipophilic agent, and this facilitates attachment to cellular membranes leading to lipid degradation and peroxidation with further release of free radicals and oxidative stress which may result in CsA-resistance and nephrotic syndrome (1, 7). I agree with Ebru Uz et al. that erdosteine may protect renal tissue by alteration of CsA induced rising oxidative stress markers i.e., glutathione peroxidase, catalase, malondialdehyde and nitric oxide levels along with histopathological changes (1). Furthermore, erdosteine may improve several toxic drug side effects such as those found in CsA, gentamicin, paracetamol, vancomycin and radiocontrast induced renal dysfunction (8, 9). On the other hand, it could also have a positive impact on other anti-oxidant enzymes i.e., xanthine oxidase and myeloperoxidase, as well as renal functional markers i.e., blood urea nitrogen, serum creatinine, uric acid, total protein, and albumin levels (2, 9). So it seems that the administration of erdosteine might be useful for the management of renal dysfunction. Erdosteine is generally well tolerated in human studies for the management of wet cough and COPD. However, mucolytics such as erdosteine are cautioned in patients with a history of peptic ulcer. As a prodrug, erdosteine may be less likely to disrupt the gastric mucosal barrier but the use of erdosteine is also contraindicated in patients with an active peptic ulcer (4).

Hence, there still remains a number of big questions to explore further; (i) possible different routes of administrating erdosteine for the management of renal failure is questionable due to an alteration in the variety of metabolic enzymes, (ii) the potential molecular mechanism of erdosteine for the beneficial effects on renal toxicity still remains unexplored, and (iii) it is clinically approved for wet cough and chronic bronchitis in humans, but relevant clinical reports for the management of renal failure still needs to be explored in human subjects. In addition, until more robust data is available the use of erdosteine cannot be recommended (10). So it is difficult to suggest the administration of erdosteine for renal failure patients because the signs and symptoms of erdosteine overlap with the health status of human subjects. However, extensive clinical studies with different doses of erdosteine are warranted in order to clarify this issue.
